# Intestinal epithelial plasticity and regeneration via cell dedifferentiation

**DOI:** 10.1186/s13619-020-00053-5

**Published:** 2020-09-01

**Authors:** Yuan Liu, Ye-Guang Chen

**Affiliations:** The State Key Laboratory of Membrane Biology, Tsinghua-Peking Center for Life Sciences, School of Life Sciences, Tsinghua University, Beijing, 100084 China

**Keywords:** Intestine, Stem cells, Regeneration, Dedifferentiation, Plasticity, Quiescence

## Abstract

The intestinal epithelium possesses a great capacity of self-renewal under normal homeostatic conditions and of regeneration upon damages. The renewal and regenerative processes are driven by intestinal stem cells (ISCs), which reside at the base of crypts and are marked by Lgr5. As Lgr5^+^ ISCs undergo fast cycling and are vulnerable to damages, there must be other types of cells that can replenish the lost Lgr5^+^ ISCs and then regenerate the damage epithelium. In addition to Lgr5^+^ ISCs, quiescent ISCs at the + 4 position in the crypt have been proposed to convert to Lgr5^+^ ISCs during regeneration. However, this “reserve stem cell” model still remains controversial. Different from the traditional view of a hierarchical organization of the intestinal epithelium, recent works support the dynamic “dedifferentiation” model, in which various cell types within the epithelium can de-differentiate to revert to the stem cell state and then regenerate the epithelium upon tissue injury. Here, we provide an overview of the cell identity and features of two distinct models and discuss the possible mechanisms underlying the intestinal epithelial plasticity.

## Background

The intestinal epithelium has three critical functions: nutrient digestion and absorption, protection and hormone secretion. It consists of nutrient-absorbing enterocytes, mucus-secreting goblet cells, hormone-producing enteroendocrine cells, defensing Paneth cells, stem cells, progenitor cells and other function less defined cells (Clevers 2013; van der Flier and Clevers 2009; Vermeulen and Snippert 2014). The intestinal epithelium is the fastest self-renewing tissue in mammals and renewed in 4–7 days, except Paneth cells with a life span of about 2 months. In the small intestine, epithelial cells are generated within invaginations of the epithelium called crypts, migrate toward the surface, and die at the tip of villi in the small intestine. Actively cycling crypt base-resident Lgr5-marked intestinal stem cells (ISCs) power this rapid cell turnover by generating daughter progenitors that are capable to undergoing multi-lineage differentiation (Barker 2014; Barker et al. 2007; Vermeulen and Snippert 2014). Wang and colleagues reported a ground intestinal stem cell type that highly express Lgr5, Olfm4, CD133 and Lrig1, and display high clonogenicity and genome stability after serial passaging in vitro (Wang et al. 2015). After they pass the crypt-villus boundary, epithelial cells become post-mitotic and mature to all functional cell types mentioned above.

**Fig. 1:**
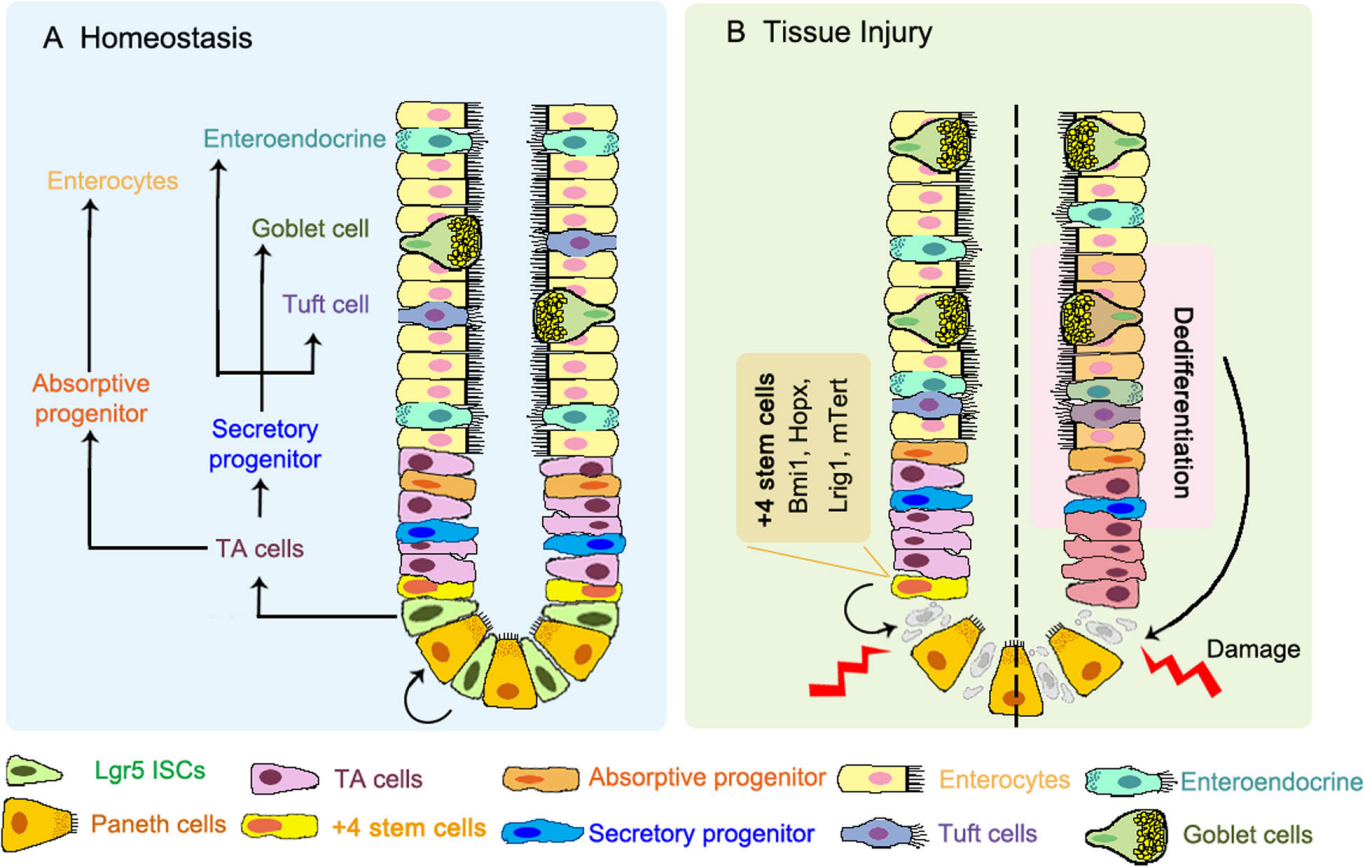
Current models of intestinal epithelium during homeostasis and regeneration. **a** During homeostasis, multipotent Lgr5^+^ ISCs maintain regular epithelial renewal. The base-locate Lgr5^+^ ISCs generate rapidly proliferating TA cells and further differentiated into the various cell lineages. **b** Tissue injury that results in the loss of Lgr5^+^ ISCs drives a regenerative response to recover the Lgr5^+^ ISC pool. Left: the reserve stem cell model: damage-resistant + 4 quiescent stem cells function as the “reserve stem cells” to refill the Lgr5^+^ pool and restore the epithelial architecture. Right: the dedifferentiation model: acute damages result in the loss of the Lgr5^+^ cells and trigger dedifferentiation of progenitors and mature cells to replenish the Lgr5^+^ ISC population

In addition to the actively proliferating Lgr5^+^ ISCs, also called crypt base columnar (CBC) cells that are required for homeostatic maintenance of the intestinal epithelium, accumulative evidence has been provided for the existence of quiescent stem cells (also called reserve stem cells) (Li and Clevers 2010; Montgomery et al. 2011; Powell et al. 2012; Takeda et al. 2011; Tian et al. 2011; Wong et al. 2012; Yan et al. 2012). The intestinal epithelium constantly faces various injuries, which rapidly destroy the actively proliferating Lgr5^+^ ISCs. The major function of the quiescent stem cells are thought to play an essential role in regeneration of the injured intestinal epithelium (Bankaitis et al. 2018), although they have also been suggested as indispensable stem cells that can give rise to all intestinal cell lineages during homeostasis (Gracz and Magness 2014). However, accumulating evidence suggests that progenitor cells or terminally differentiated cells can also re-enter the cell cycle and regain the stem cell activity to regenerate the damaged epithelium (Buczacki et al. 2013; de Sousa and de Sauvage 2019; van Es et al. 2012; Yan et al. 2017; Yu et al. 2018) (Fig. 1).

## Quiescent stem cells

Quiescent stem cells have the properties of self-renewal, multi-potentiality and long-term label retaining capacity, but lack cell proliferation markers (Fuchs 2009). PCNA, Ki67, and phospho-Histone H3 have been widely used as proliferating cell markers. As quiescent stem cells are not actively proliferating, they cannot be labeled with these proliferation markers (Barriga et al. 2017; Buczacki et al. 2013; Li et al. 2016; Roche et al. 2015; Yu et al. 2018). Furthermore, these markers can only be labeled in the fixed cells or tissues, owing to formaldehyde and permeable treatment.

Several approaches have been employed to identify quiescent stem cells. A common approach is to label the cells with nucleotide analogs such as 5-bromo 2′-deoxyuridine (BrdU) and 5-ethynyl-2′-deoxyuridine (EdU) and then following label retention over the time. A pulse treatment of BrdU or EdU can efficiently label dividing cells. Quiescent stem cells could retain the labels for a long time, while the labels in proliferating cells would be diluted after each division and disappear eventually (Roche et al. 2015; Yu et al. 2018). These quiescent cells are also regarded as “label-retaining cells” (LRCs) (Barriga et al. 2017; Buczacki et al. 2013; Li et al. 2016). A limitation of the EdU/BrdU label is that these labels can only be identified after staining with antibody, and living quiescent stem cells cannot be directly observed. To overcome this limitation, chromatin labels with histone 2B (H2B) fused with a fluorescent protein (such as GFP or YFP) were utilized (Buczacki et al. 2013). Similar with DNA labels, quiescent stem cells retaining H2B-GFP/YFP for an extended time could be observed directly in vivo and allow subsequent characterization of these cells (Barriga et al. 2017).

Besides DNA labeling and chromatin labeling, lineage-tracing has been used to characterize quiescent or slowly cycling ISCs and verify their self-renewal and multi-potentiality (Asfaha et al. 2015; Barriga et al. 2017; Montgomery et al. 2011; Sangiorgi and Capecchi 2008). Upon tissue damage, for instance, due to irradiation and inflammation, quiescent stem cells can be activated and revert to the actively proliferating Lgr5^+^ ISCs to re-establish the epithelium architecture. Lineage tracing can follow up this process in vivo to mark the progenies derived from labeled quiescent stem cells. Several markers of quiescent stem cells have been used for lineage tracing, such as Bmi1 (Sangiorgi and Capecchi 2008; Tian et al. 2011) and Hopx (Takeda et al. 2011).

Recent development of single-cell mRNA-seq (scRNA-seq) technology has greatly facilitated the investigation on cell identity, heterogeneity, lineage hierarchy, and the identification of prospective stem cell markers (Ayyaz et al. 2019; Kim et al. 2016; Tetteh et al. 2016). Using H2B-GFP mouse model in combination with scRNA profiling, Li et al. found that most LRCs are in the G1 phase and can be divided to short-term LRCs (label-retained 10 days after pulse) with secretory cell markers and long-term LRCs (label-retained over 1 month after pulse) with Paneth cell markers, whereas Hopx-marked reserve ISCs are primarily quiescent in G0 (Li et al. 2016). Although some of the short-term LRCs are also in G0, but they are enteroendocrine-like and Hopx-negative. Therefore, they concluded that LRCs are distinct from reserve ISCs. Similarly, single-cell transcriptomic profiling has successfully employed to identify new markers or new types of quiescent ISCs. For instance, Mex3a is shown to mark a subpopulation of Lgr5^+^ ISCs that can generate all intestinal lineages (Barriga et al. 2017); Clusterin marks a class of the cells that are rare and distinct from Lgr5^+^ cells, and these cells are greatly expanded upon injuries via a YAP-dependent manner and play an essential role in the epithelial regeneration (Ayyaz et al. 2019). Furthermore, scRNA-seq analysis revealed that Bmi1^+^ cells are enriched for enteroendocrine markers, while Prox1 marked both enteroendocrine cells and tuft cells (Yan et al. 2017). Both Bmi1^+^ and Prox1^+^ cells are capable to convert to the ISC state during homeostasis and injury-induced regeneration (Yan et al. 2017). More recently, scRNA-seq-based pseudotime analysis revealed that regenerating Ascl2^+^ cells are originated from the absorptive or secretory lineage and undergo dedifferentiation, eventually gain the ISC characteristics and regenerate the damaged epithelium (Murata et al. 2020).

Several markers have been described for quiescent ISCs, such as Bmi1, Tert, Hopx and Lrig1. The most recognized quiescent ISCs are + 4 LRCs, which reside at the position + 4 from the base of crypts, retain DNA labels for long time due to their slow cycling activity and are resistant to high doses of irradiation (Roth and Fodde 2011; Scoville et al. 2008). However, several other cell types, like Krt19^+^ cells and Lgr5^high^ cells, have also been suggested to possess quiescent stem cell properties and share the quiescent stem cell markers (see the following).

### Bmi1

The concept of + 4 stem cells came from Hendry and Potten in 1974 (Hendry and Potten 1974), while the first identified marker of these cells is Bmi1, a key component of polycomb repressive complex 1. By in situ hybridization and lineage tracing, the Bmi1 was detected in the cells located near the bottom of crypts in the small intestine, predominantly + 4 position from the base, and could proliferate, expand and give rise to all cell lineages of intestinal epithelium (Lopez-Arribillaga et al. 2015; Sangiorgi and Capecchi 2008; Tian et al. 2011). Further studies unraveled that the Bmi1^+^ cell population is functionally distinct from Lgr5^+^ ISCs and characterized as quiescent ISCs (Tian et al. 2011; Yan et al. 2012). The results from irradiation or Diphtheria toxin-mediated depletion of Lgr5^+^ ISCs support the “reserve stem cell model”, in which Lgr5^+^ ISCs mediate homeostatic self-renewal, whereas Bmi1^+^ + 4 ISCs refill the Lgr5^+^ ISC pool and regenerate the damaged epithelium upon injuries (Tian et al. 2011; Yan et al. 2012).

However, later studies revealed that Bmi1 expression is not restricted to + 4 cells, but also in other crypt cells and some differentiated cells (Itzkovitz et al. 2011; Jadhav et al. 2017; Lopez-Arribillaga et al. 2015; Munoz et al. 2012; Yan et al. 2017). For instance, Bmi1 expression was found in Dll1^+^ secretory progenitors (van Es et al. 2012). Bmi1-GFP^+^ cells also express mature enteroendocrine cell markers, including Chga, Neurod1 and Prox1 (Jadhav et al. 2017; Yan et al. 2017). Collectively, all these studies indicate that Bmi1 is not a good marker of reserve ISCs. Mechanistically, Bmi1 is important to sustain proliferation of the Bmi1^+^ ISCs and progenitors in the crypt, likely by repressing the expression of p16^INK4a^ and p19^ARF^ (Lopez-Arribillaga et al. 2015).

### Hopx

The homeodomain transcription factor Hopx was originally proposed to mark + 4 quiescent ISCs by lineage tracing, and it is proposed that the two ISC populations, active Lgr5^+^ CBCs and Hopx^+^ reserve ISCs, can interconvert to each other in the small intestine (Takeda et al. 2011). Later, Hopx was shown to label a type of quiescent stem cells, named Hopx^+^ colitis-associated regenerative stem cells (Hopx^+^ CARSCs) in colon, which contribute to mucosal regeneration during dextran sulfate sodium (DSS)-induced colitis (Wang et al. 2019). Interestingly, distinguished from the slow-cycling Hopx^+^ stem cells that display a constant Hopx expression identified by Takeda et al., Hopx^+^ CARSCs display a transient discontinuation of Hopx expression upon injury and then proliferate to reestablish the colon architecture. The secretory progenitor cells marked by the transcription factor Atoh1 have also been shown to repopulate the colonic epithelium during DSS-induced colitis (Castillo-Azofeifa et al. 2019; Ishibashi et al. 2018; Tomic et al. 2018). Therefore, it would be interesting to know if Atoh1^+^ progenitors overlap with Hopx^+^ CARSCs. It is worth noting that Hopx was reported to express broadly in all proliferative crypt cells including Lgr5^+^ ISCs (Munoz et al. 2012; Powell et al. 2012), suggesting that it is not a suitable marker for reserve ISCs.

### Tert

Telomerase reverse transcriptase (Tert) labels LRCs that are functionally distinct from Lgr5^+^ ISCs in the intestinal epithelium (Breault et al. 2008), suggesting that it can mark reserve ISC population. Consistently, Tert^+^ cells were found to regenerate all types of differentiated cells as well as Lgr5^+^ stem cells upon irradiation (Montgomery et al. 2011).

### Lrig1

EGF stimulates ISC proliferation via the EGFR/MAPK pathway (Sato et al. 2009). The transmembrane protein Lrig1 can interact with the EGFR family members and recruit the E3 ubiquitin ligase c-CBL to promote EGFR degradation and thus constrain EGF signaling (Wang et al. 2013). Lgri1 has been shown to mark a subset of ISCs that occupy in the crypts base and are relatively non-cycling under homeostasis, and these cells rapidly divide to re-establish damaged crypts upon tissue injury (Powell et al. 2012). However, another study showed that Lrig is highly expressed in actively proliferating Lgr5^+^ ISCs (Wong et al. 2012). As Lrig1 expression is induced by EGF signaling, it is proposed that Lrig1 controls the size of the stem cell niche in a negative-feedback manner (Wong et al. 2012).

### Mex3a

The RNA-binding protein Mex3a has also been reported to define LRCs. The Mex3a^+^ cells are a slowly proliferating subpopulation of Lgr5^+^ stem cells and occupy the + 3/+ 4 crypt position (Barriga et al. 2017). Using the H2B-YFP mouse model and lineage tracing, Barriga et al. demonstrated that Mex3a^high^ cells displayed slowly cycling characteristics and could generate crypt-villus ribbons albeit at an infrequent rate under the homeostatic condition. Upon damage by 5-FU treatment or ionizing radiation, Mex3a^high^ Lgr5^+^ cells are largely reserved, and can refill the ISC pool and generate the epithelium. Besides, single-cell transcriptomic analysis demonstrated that two classes of Lgr5^high^ cells, Mex3a^+^ and Mex3a^−^, co-exist in the crypts. In line with the idea that Mex3a^high^ cells are LRCs, they are enriched in BMI1, Lrig1, Tert and Hopx and display a low proliferation. A recent study also confirmed the role of Mex3a in the maintenance of the Lgr5^+^ ISC pool and further showed that it does so possibly by suppressing PPARγ signaling (Pereira et al. 2020). PPARγ-selective agonists reduce the number and size more markedly in Mex3a KO organoids than WT organoids, which are consistent with the observation that Mex3a downregulates PPARγ expression and high PPARγ signaling impairs Lgr5^+^ ISCs (Pereira et al. 2020).

In addition to these markers, other + 4 cell markers were reported based on their expression in rare subpopulation (Barker et al. 2012), for instance, Pten (Bjerknes and Cheng 2005; He et al. 2007), Dclk1 (May et al. 2008), and WIP1 (Demidov et al. 2007). However, None of these markers are specifically restricted in + 4 cells. For instance, Krt19^+^ cells are found enriched with the + 4 ISCs markers Bmi1, Hopx and Lrig1, as well as Dll1 (Asfaha et al. 2015). Nearly all Lgr5^high^ cells co-express high levels of the reserve ISCs markers Bmi1, Lrig1 and Hopx (Munoz et al. 2012; Roche et al. 2015). Therefore, although quiescent intestinal stem cells have been described for the past decade, the identity and features of such cells remain under debate.

## Intestinal epithelium plasticity and cell dedifferentiation

Lgr5^+^ CBCs are well documented to be responsive for the homeostatic renewal of the intestinal epithelium (Clevers 2013), although they have also been shown to be essential for repair of the irradiation-damaged epithelium (Metcalfe et al. 2014). Nonetheless, the intestinal epithelium exhibits a great plasticity after the injury-mediated elimination of Lgr5^+^ ISCs. In response to epithelium injuries or perturbations, several types of progenitors and some fully differentiated cells have been shown to possess the dedifferentiation ability to generate the actively cycling Lgr5^+^ stem cells and contribute to the regeneration of the damaged epithelium (de Sousa and de Sauvage 2019). These cells include label-retaining secretory precursors (Buczacki et al. 2013; Li et al. 2016), Alpi^+^ enterocyte precursors (Tetteh et al. 2016), Dll1^+^ progenitors (van Es et al. 2012), Atoh1^+^ progenitors (Ishibashi et al. 2018; Tomic et al. 2018), enteroendocrine cells (Yan et al. 2017), tuft cells (Westphalen et al. 2014) and Paneth cells (Schmitt et al. 2018; Yu et al. 2018). In addition, the intermediate filament keratin-19 (Krt19) has been shown to mark long-lived, radiation-resistant cells at upper crypts, and these cells can undergo dedifferentiation and regenerate Lgr5^+^ ISCs in both the small intestine and colon (Asfaha et al. 2015). However, the Krt19^+^ cells may be heterogeneous rather than represent a specific cell population.

### Secretory progenitors

Secretory progenitors differentiate to Paneth cells, goblet cells, enteroendocrine cells and tuft cells. Several studies have highlighted the plasticity and ISC-oriented dedifferentiation of secretory precursors. The cells marked with the Notch ligand Dll1 can differentiate to those mature secretory cells at a low frequency during homeostasis, but these Dll1^+^ cells undergo dedifferentiation and refill the stem cell pool upon irradiation-induced injury (Murata et al. 2020; van Es et al. 2012). Three types of Atoh1^+^ secretory progenitors, including Atoh1^+^ Lgr5^+^ (Kim et al. 2016), Atoh1^+^ LRC (Buczacki et al. 2013), and Atoh1^+^ Dll1^+^ (van Es et al. 2012), contribute to multilineage of secretory cells in a higher frequency than Dll1^+^ cells in the steady state. These Atoh1^+^ cells can also repair and replenish the colonic epithelium during DSS-induced colitis (Ishibashi et al. 2018; Tomic et al. 2018).

Using H2B-YFP to identify label-retaining cells, Buczacki and colleagues showed that LRCs are located throughout the crypt base, but not specifically at the position + 4, and these cells could contribute to the stem-cell pool and repopulate the epithelium after damage (Buczacki et al. 2013). RNA expression profiling revealed that these non-Paneth LRCs express not only Lgr5 but also quiescent stem-cell markers Tert, Lrig1 and Hopx, representing a subset of Lgr5^+^ cells that are able to differentiate to Paneth and enteroendocrine cells. In addition, the transcription factor Sox9, which is required for Paneth cell differentiation (Bastide et al. 2007), can also label the reserve ISCs that are resistant to irradiation and express Bmi1, Lrig1, Hopx and Dll1 (Furuyama et al. 2011; Roche et al. 2015). Interestingly, these Sox9^high^ cells also express Lgr5 and may represent secretory progenitors. Of note, although Sox9 ablation in the intestinal epithelium leads to loss of regenerative capacity and increased apoptosis after irradiation (Roche et al. 2015), it is unclear whether the regeneration defect is due to the loss of reserve ISCs as Sox9 are also expressed in other types of cells and required for Paneth cell differentiation. The same concern also applies to other cases in which a marker is not specific but labels multi-types of cells.

### Absorptive progenitors

By following the fate of the enterocytes labeled by alkaline phosphatase (Alpi), Tetteh et al. observed that some of Alpi^+^ enterocytes can display de-differentiation and generate the crypt-villus “ribbons” upon Lgr5^+^ ISC ablation with diphtheria toxin in *Alpi*^*CreER*^***;****R26R*^*LacZ*^***;****Lgr5*^*DTR-GFP*^ mouse model (Tetteh et al. 2016). The dedifferentiating Alpi^+^ enterocytes should represent enterocyte progenitors as the Alpi^+^ cells lose the dedifferentiation capacity after exit from the crypt. Although the detailed mechanism underlying the dedifferentiation is unclear, sc-RNA profiling uncovered that the dedifferentiating Alpi^+^ cells express the genes involved in proliferation and stemness, and interestingly share some Paneth cell markers.

### Enteroendorine cells

As discussed above, Bmi1 is regarded as a quiescent stem cell marker previously (Sangiorgi and Capecchi 2008; Tian et al. 2011; Yan et al. 2012), but later bulk RNA-seq and scRNA-seq unveiled that it is not specifically expressed in quiescent cells (Itzkovitz et al. 2011; Jadhav et al. 2017; Munoz et al. 2012; van Es et al. 2012; Yan et al. 2017). Instead, Bmi1^+^ cells display a gene expression signature associated with mature enteroendocrine cells that can revert to Lgr5^+^ ISCs and regenerate the intestinal epithelium after ISC loss (Jadhav et al. 2017; Yan et al. 2017). Of note, Bmi1^+^ enteroendocrine cells that undergo injury-induced dedifferentiation are also marked by the transcription factor Prox1 (Yan et al. 2017).

### Paneth cells

Differentiated Paneth cells could also provide an alternative for regeneration after injury. Yu et al. demonstrated that mature Paneth cells, marked by Lyz1^+^, could re-enter the cell cycle to repopulate the epithelium in response to irradiation (Yu et al. 2018). The dedifferentiation process depends on Notch signaling, but not Wnt/β-catenin signaling. Moreover, mature Paneth cells have been demonstrated to re-enter the cell cycle and regenerate the whole intestine epithelium upon inflammation (Schmitt et al. 2018). However, different mechanisms were proposed – inflammation stimulates the expression of stem cell factor (SCF) which activates β-catenin via c-Kit/Akt signaling and then induces the cell cycle re-entry of Paneth cells. Therefore, different mechanisms may account for different stimuli to activate the dedifferentiation processes of even the same type of cells.

### Tuft cells

Doublecortin like kinase 1 (Dclk1, also known as doublecortin and CaM kinase-like 1 (DCAMKL-1), was reported as a possible reserve stem cell marker in the intestine (May et al. 2008; May et al. 2009), but it is also found enriched in tuft cells in the stomach and intestine (Gerbe et al. 2009; Saqui-Salces et al. 2011; Westphalen et al. 2014). Westphalen et al. showed that a small population of Dclk1^+^ cells could display infrequent formation of crypt-villus ribbons under homeostasis (Westphalen et al. 2014). Furthermore, ablation of Dclk1^+^ tuft cells revealed that these differentiated cells contribute to regeneration of the intestinal epithelium upon irradiation or DSS-induced injury. Interestingly, when Dclk1^+^ tuft cells harbor APC mutation, inflammatory stimulation, these cells display the tumor-initiating ability. These data together indicate that a subset of Dclk1^+^ tuft cells are long-lived, retain the ability to dedifferentiate and regenerate the intestinal epithelium, or form tumors in loss of APC function upon injury (Westphalen et al. 2014). Furthermore, in addition to labeling the Bmi1^+^ cells with a mature enteroendocrine phenotype, Prox1 also marks a subset of tuft cells that can contribute to intestinal epithelium homeostasis and irradiation-induced regeneration (Yan et al. 2017).

In addition to the cell types discussed above, CD69^+^CD274^+^ goblet cell precursors have been shown to repopulate the ablated Lgr5+ ISCs and contribute to epithelial regeneration after injuries (Jadhav et al. 2017).

## Regulation of cell plasticity

In the homeostatic intestinal epithelium, the hierarchical differentiation processes from the stem cells to the mature functional cells are tightly controlled by niche factors (Barker 2014; Clevers 2013; Medema and Vermeulen 2011; Qi and Chen 2015). Wnt/β-catenin signaling is essential for the maintenance of ISCs’ self-renewal and proliferation as well as Paneth cell differentiation (van Es et al. 2012; Yin et al. 2014); BMP/Smad signaling counteracts Wnt signaling to block ISC stemness and promotes enterocyte differentiation, as well as inducing cytostatic effects and cell death (Wang and Chen 2018); EGF/MAPK signaling stimulates ISC proliferation (Sato et al. 2009), although it is dispersible in the in vitro Lgr5^+^ stem cell maintenance in the presence of the GSK3 inhibitor CHIR99021 and the BMP receptor inhibitor LDN-193189 (Li et al. 2018). Notch signaling displays a different effect on ISCs – it promotes self-renewal in the presence of high Wnt signaling activity while boosting the enterocyte lineage under conditions of Wnt inhibition (Medema and Vermeulen 2011; Yin et al. 2014). Conversely, Notch inhibition enhances secretory lineage specification (Yin et al. 2014). However, it is less clear what signals modulate the regeneration processes under injury conditions. Much attention has been paid to identification of the quiescent stem cells that are activated upon injuries or the differentiated cells that undergo dedifferentiation to replenish the damage-depleted stem cell pool and regenerate the epithelium. Despite it, some of works have shed light on the mechanisms underlying the regeneration process.

Several intrinsic factors have been implicated in cell plasticity control. ATOH1 marks secretory progenitors that are able to revert to stem cells upon injuries (Ishibashi et al. 2018; Tomic et al. 2018). Further studies revealed that the cyclin-dependent kinase-mediated phosphorylation of ATOH1 at multiple sites is required for the conversion of secretory progenitors to stem cells and then for epithelial regeneration as knockin of the phosphorylation-defective mutant Atoh1 promotes secretory differentiation with the compromised epithelium proliferation and prevents injury-induced regeneration in mice (Tomic et al. 2018). The basic helix-loop-helix transcription factor Ascl2 plays a critical role in regulating of the actively cycling Lgr5^+^ ISC pool (Schuijers et al. 2015; van der Flier et al. 2009). Recently, Murata et al. showed a critical role of Ascl2 during regeneration (Murata et al. 2020). Depletion of Lgr5^+^ ISCs leads to Ascl2 expression in the absorptive and secretory precursors located in the middle crypts region, and these cells then dedifferentiate to Lgr5^+^ ISCs and regenerate the damaged epithelium (Liu, 2020). One of Ascl2-regulated genes is interleukin (IL) 11 receptor IL11RA1 that may be involved in the regeneration process. Although Ascl2 is a Wnt target (van der Flier et al. 2009), it remains unclear what signals induce its expression in the absorptive and secretory precursors upon injuries. Moreover, using the air-liquid interface 2D culture system to mimic the injury-regeneration process, hypoxia and endoplasmic reticulum stress have been indicated involved in the Hopx^+^ CARSCs-mediated regeneration of the intestinal epithelium upon inflammation (Wang et al. 2019). The RNA-binding protein Msi, which are expressed throughout the crypts, has been shown to drive the exit of quiescent stem cells from G0 into the cell cycle upon irradiation (Yousefi et al. 2016).

Chromatin accessibility analysis revealed that chromatin access undergoes a dynamic change in a group of enhancer regions: they remain open during the differentiation of Lgr5^+^ ISCs to secretory cells and quickly close up during the dedifferentiation of Bmi1^+^ and CD69^+^CD274^+^ cells to replenish the Lgr5^+^ ISC pool (Jadhav et al. 2017). In line with the chromatin reorganization to change gene expression profiling, several studies have uncovered the induction of a fetal signature in the regenerative colonic crypts with the DSS mouse model (Nusse et al. 2018; Wang et al. 2019; Yui et al. 2018).

Extrinsic factors produced by the microenvironment also participate in cell reprogramming and epithelial regeneration. As discussed above, different mechanisms have been suggested to induce Paneth cell dedifferentiation: β-catenin activation via the SCF/c-Kit/Akt signaling axis is critical for the inflammation-provoked cell-cycle re-entry of Paneth cells (Schmitt et al. 2018), while Notch signaling, but not Wnt/β-catenin signaling, is important for Paneth cells to acquire stem cell features upon irradiation (Yu et al. 2018). YAP/TAZ signaling was suggested to be important for ISC regeneration (Gregorieff et al. 2015; Nusse et al. 2018; Yui et al. 2018). Yui et al. have linked the extracellular matrix signals to regulate inflammation-triggered regeneration of the intestinal epithelium (Yui et al. 2018). Collagen can activate YAP/TAZ via FAK/Src signaling, and YAP/TAZ then promotes cell reprogramming and tissue regeneration. This axis may also operate as mechano-sensing pathway (Yui et al. 2018). Furthermore, Basak et al. showed that inhibition of EGFR/ERK signaling can convert actively proliferating Lgr5^+^ ISCs to quiescent Lgr5^+^ ISCs with the enteroendocrine cell signature (Basak et al. 2017).

Moreover, the non-epithelial niche also contributes to regeneration. IL-22 produced by innate lymphoid cells (ILCs) can promote Lgr5^+^ ISC expansion and epithelial regeneration (Lindemans et al. 2015). But this observation was challenged by a recent study, which showed that IL-22 expands TA cell population and meanwhile reduce Lgr5^+^ ISCs by inhibiting Wnt signaling and Notch signaling (Zha et al. 2019). Besides immune cells, a sub-population of GLI1^+^ mesenchymal cells are enriched during regeneration following DSS-induced colonic damage, and these cells may provide R-spondin3 to facilitate epithelial damage repair (Degirmenci et al. 2018). Together, these studies support a crucial role of the niche near the base of crypts for regenerative potential of the intestine.

## Perspectives

The intestinal epithelium is a great system to investigate homeostatic maintenance due to its fast turnover, relatively simple structure, the identification of Lgr5^+^ stem cells and the well-documented differentiation processes to the known cell lineages. Much evidence also indicates that the intestinal epithelium has a great regenerative plasticity to repair the damaged lesions after the normal Lgr5^+^ stem cell pool is ablated by injuries. In the past few years, identification of the cells responsible for regeneration is under extensive investigation. Label-retaining experiments suggested that the cells at the position 4 of the crypt base are the quiescent stem cells that are activated upon injuries and responsive for the epithelial regeneration (Roth and Fodde 2011; Scoville et al. 2008). A major challenge in this field is posed by the fact that expression of the markers traditionally used to label the quiescent stem cells is not restricted to a given cell type. In fact, the expression of many proposed markers are overlapped in actively cycling Lgr5^+^ ISCs, quiescent stem cells, progenitors and mature cells (Asfaha et al. 2015; Barriga et al. 2017; Munoz et al. 2012; Powell et al. 2012; Roche et al. 2015; van Es et al. 2012; Yan et al. 2017). This raises a critical question to the “reserve stem cell” regeneration model. Recently, emerging evidence supports the “dedifferentiation” model, which suggests that partially or even fully differentiated cells undergo dedifferentiation to replenish the Lgr5^+^ ISC pool and then drive the intestinal epithelial regeneration.

By far, almost all the lineage progenitors and maturely differentiated cells have been demonstrated to re-enter cell cycle, regain stemness and replenish the epithelium upon injuries (Table 1). Despite the exciting progress, some key questions remain to be addressed. For instance, the actively cycling Lgr5^+^ ISCs are most vulnerable to damages and are the first ablated by injuries. How is their absence sensed to trigger quiescent stem cell activation or de-differentiation of the committed progenitors or mature cells into stem cells? What signals are involved? It has been shown that actively cycling ISCs and quiescent stem cells can convert to each other (Barriga et al. 2017; Takeda et al. 2011). Then what controls this conversion? Moreover, three types of mouse models have been commonly employed to study intestinal epithelial regeneration: DTR-based ablation of Lgr5^+^ cells, irradiation and DSS-based inflammation. It is unclear how these distinct injuries impact on the cell types to repopulate the Lgr5^+^ cell pool and the underlying mechanisms. In another word, different types of injury may activate different regeneration mechanisms. Another important question is whether the mechanisms found in mice can apply to humans, in particular by considering that multiple types of inflammatory bowel disease are found in humans.

**Table 1:** The intestinal cells capable of self-renewal or de-differentiation

Marker	Lineage	Overlapping expression	Tissue	Damage model	Mechanism	Reference
Bmi1	+4 reserve stem cells	Lgr5, Prox1, mTert, Krt19	Small Intestine	Irradiation, ablation of Lgr5+ through Lgr5-DTR mice	Unclear	(Lopez-Arribillaga et al. 2015; Sangiorgi and Capecchi 2008; Tian et al. 2011; Yan et al. 2012)
	Enteroendocrine cells	Prox1	Small Intestine	Irradiation	Open chromatin	(Jadhav et al. 2017; Yan et al. 2017)
Hopx	+4 reserve stem cells	Lgr5, mTert, Krt19	Small Intestine	–	Unclear	(Takeda et al. 2011)
	Regenerative stem cells	ATOH1	Colon	DSS-induced colitis	Oxygen tension	(Wang et al. 2019)
Lrig1	Active ISCs	Lgr5	Small Intestine	–	Negative regulation of ErbB signaling	(Wong et al. 2012)
	+4 reserve stem cells	Lgr5, Hopx, mTert, Krt19	Small intestine and colon	Irradiation	Negative regulation of ErbB signaling	(Powell et al. 2012)
Tert	+4 reserve stem cells	Lgr5, Bmi1, Mex3a	Small Intestine	Irradiation	Unclear	(Barriga et al. 2017; Breault et al. 2008; Montgomery et al. 2011)
Dclk1	+4 reserve stem cells	Msi1	Small Intestine	Irradiation	Unclear	(May et al. 2008; Nakanishi et al. 2013)
	Tuft cells	–	Small intestine and colon	Irradiation, DSS-induced colitis	Unclear	(Gerbe et al. 2009; Westphalen et al. 2014)
LRCs^a^	Secretory progenitors (short-term)	Lgr5, Hopx, Dll1, ATOH1, Sox9, Krt19	Small Intestine	Irradiation	Unclear	(Buczacki et al. 2013; Li et al. 2016; Roche et al. 2015)
	Paneth and Enteroendocrine -like cells (long-term)	Lyz1, c-Kit	Small Intestine	Irradiation	Unclear	(Li et al. 2016; Roche et al. 2015)
Dll1	Secretory progenitors	Sox9, ATOH1, Krt19	Small Intestine	Irradiation	Unclear	(van Es et al. 2012)
ATOH1	Secretory progenitors	Lgr5, Dll1, Hopx	Small intestine and colon	Irradiation, DSS-induced colitis	Multisite phosphorylation of ATOH1	(Ishibashi et al. 2018; Tomic et al. 2018)
Sox9	LRCs	Lgr5, Bmi1, Hopx, Lrig1, Dclk1, Dll1	Small Intestine	Irradiation	Unclear	(Roche et al. 2015)
Krt19	Upper crypts	Lgr5, Bmi1, Hopx, Lrig1, mTert, Dll1	Small intestine and colon	5-FU, ablation of Lgr5+ through Lgr5-DTR mice	Unclear	(Asfaha et al. 2015)
Lyz1	Paneth cell	c-Kit	Small Intestine	Irradiation, inflammation	SCF/c-Kit/Wnt pathway, forced activation of Notch pathway	(Schmitt et al. 2018; Yu et al. 2018)
Alpi	Enterocyte progenitor	–	Small Intestine	Ablation of Lgr5+ through Lgr5-DTR mice	Unclear	(Tetteh et al. 2016)
CD69, CD274	Goblet cell progenitor	–	Small Intestine	Ablation of Lgr5+ through Lgr5-DTR mice	Open chromatin	(Jadhav et al. 2017)
Prox1	Enteroendocrine cells, tuft cells	Bmi1	Small Intestine	Irradiation	Unclear	(Yan et al. 2017)
Mex3a	Quiescent stem cells	Lgr5	Small Intestine	5-FU, ionizing radiation	Unclear	(Barriga et al. 2017)
Ascl2	Enterocyte progenitor and secretory progenitor	Smoc2, Cdca7	Small intestine and colon	Ablation of Lgr5+ through Lgr5-DTR mice	Ascl2 to activate expression of its targets such as Il11ra1	(Murata et al. 2020)
Sca1	Repairing epithelium	Lrig1	Colon	DSS-induced colitis	Reprogram into fetal-like state by activation of YAP/TAZ pathway	(Yui et al. 2018)
	Progeny of ISCs	–	Small intestine	Infection by H.polygyrus, irradiation, ablation of Lgr5+ through Lgr5-DTR mice	IFN-γ-denpendent fetal-like transcriptional program	(Nusse et al. 2018)

^a^*LRCs* Nucleotide analog label-retaining cells

New methodologies to study stem cell quiescence in vivo need to be developed eagerly. In many studies, the identification of quiescent ISCs was primarily based on single biomarkers. However, these markers are broadly expressed within the crypt, even in the villus (Munoz et al. 2012; Yan et al. 2017), limiting their reliability and the lineage tracing based on them to define the cell population. Furthermore, various cell isolation methods such as fluorescent-activated cell sorting (FACS) are used for cell identity validation and function studies. However, these methods may impact the cell behavior and their gene expression profile due to cell junction disruption and departure from their niche. Therefore, better strategies that would avoid additional injuries and are able to acquire profiling in situ without isolation are badly needed. In addition, it has become increasingly clear that metabolism, including oxidative phosphorylation (OXPHOS), glycolysis, fatty acid oxidation, and ketone body signaling, could affect the fate of stem cells under both physiological and pathological conditions (Berger et al. 2016; Beyaz et al. 2016; Cheng et al. 2019; Mah et al. 2014; Mihaylova et al. 2018; Rath et al. 2018; Rodriguez-Colman et al. 2017; Schell et al. 2017; Stine et al. 2019). Fasting also enhances intestinal stem cell function during homeostasis and aging, perhaps by activating fatty acid oxidation (Mihaylova et al. 2018). Furthermore, the dietary-responsive phospholipid-cholesterol axis regulates ISC proliferation (Wang et al. 2018). More recently, Morral et al. discovered that zonation of rDNA/protein synthesis could define a stem cell hierarchy colorectal cancers (Morral et al. 2020). Quiescent stem cells have been indicated to be in the minimal metabolic requirements (Signer et al. 2014; Simsek et al. 2010). It would be interesting to explore if combining the above data and proliferation information facilitates the identification of quiescent stem cells or dedifferentiating cells.

The regeneration capability is essential for maintenance of the homeostatic function of the intestinal epithelium. Deregulation of the regeneration process leads to gut pathogenesis. Therefore, delineation of the involved factors/cells would provide a better understanding about the intestinal regeneration. It will also allow us to manipulate the stem cell state and explore promising therapeutic strategies to treat colorectal cancer and inflammatory bowel diseases.
